# Announcing the new launch of *Neurological Research and Practice*

**DOI:** 10.1186/s42466-019-0011-7

**Published:** 2019-02-28

**Authors:** Werner Hacke

**Affiliations:** 1 Neurology, University of Heidelberg, Im Neuenheimer Feld 400, 69120 Heidelberg, Germany; 2 Neurological Research and Practice, Heidelberg, Germany

Welcome to the first set of articles from the new open access journal *Neurological Research and Practice*. This journal is published by the German Neurological Society (Deutsche Gesellschaft für Neurologie, DGN) in partnership with BMC, a part of Springer Nature.

You may wonder whether there is a need for another neurology journal, given the continuously growing number of options available in which to publish your research.

*Neurological Research and Practice* is different from other new open access journals. Firstly, this journal is owned and edited by the German Neurological Society and not by a commercial publishing company. Secondly, although we will provide open access, there will be no costs for authors in the first few years of establishing the journal; all costs will be covered by the DGN. Thirdly, this is the first and only English language publication by the DGN providing easy access to the broad spectrum of scientific activities coming from German neurology, especially the broad range of high quality work that just misses being published in the highest ranked general medicine journals or neuroscience specialty publications.

Historically, German neurology was connected to three German language journals; one of them was the „Zeitschrift Neurologie “(which became the Europe-based Journal of Neurology (J Neurol) some 30 years ago), the journal *Der Nervenarzt* (Springer Medizin, founded in 1928) still exists today and Aktuelle Neurologie (Thieme) was closed at the end of 2018. In 2018 another society owned, bimonthly German language publication called *DGNeurologie* was launched by the DGN.

The board of the DGN had discussed, for some time, the publication of an electronic-only open access English language journal to make the role and the achievements of German neurology more visible to an international audience. Although research papers from German neurologists are frequently found in leading international publications, in fact, most highly ranked journals frequently feature articles from German authors. Journal articles from German authors are among the most highly cited publications found in many main topics of modern neurology and neuroscience, such as stroke, neuroimmunology, epilepsy, headache, movement disorders, neuro-oncology and neurogenetics. Still, the actual role of German institutional and scientific neurology is not easily accessible to international readers. Not many experts know that the DGN has almost 10,000 active members and thereby is the second largest neurological society worldwide. Our annual congress attracts more than 7000 German-speaking neurologists and is also one of the largest national neurology conferences. In Germany neurology has evolved into the fourth largest medical specialty, has become very popular with young physicians and is one of the few medical disciplines in Germany that produce positive budgets.

With that in mind, the board of the DGN decided to take the opportunity to launch the new journal, a decision that dates back a little more than a year. Since then, we have agreed on the scopes of the journal, the article types we want to publish, have created a large international Editorial Board representing all areas of clinical and basic neurosciences including also neurosurgical, neuroradiological, neuropathological, and neuropediatric expertise.

*Neurological Research and Practice* will feature review articles, research articles, and clinical trial protocols, which will all undergo rigorous single-blind peer review. In addition we will feature abridged English versions of official guidelines approved by the DGN. Another special article type featured in the journal will be the publication of Standard Operating Procedures (SOP) that will allow a quick and comprehensive overview of state-of-the-art decision making. Finally, we will also feature articles dealing with historical aspects of German neurology in general and in specific areas of interest to German neurology, such as critical care neurology, palliative care neurology, neuro-ultrasound and others.

As the Editor-in-Chief I have appointed section editors for guidelines (Dr Hans Christoph Diener and Dr. Christian Weimar), for SOP (Dr Gereon Fink and Dr. Julian Boesel) and for history of neurology (Dr Martin Grond and Dr. Axel Karenberg). As the journal grows, more topical section editors will be appointed.

I am delighted to present the first articles to be published in *Neurological Research and Practice*, running exactly to the schedule we had planned a year ago. As an old fashioned researcher and writer I am still used to the historical aspect of planning and collecting content to publish editions of a journal unlike the continuous publication model where articles are published monthly, as they are ready, which is our ultimate aim for this new journal. For this inaugural edition we have followed that tradition: you will find a spectrum of all types of articles and a broad view of different areas in neurology. This first ‘edition’ includes one review article on „Navigating choice in multiple sclerosis management“, two research articles dealing with the „Uptake of recanalizing treatment for stroke in Germany“ and an article from the US on „GCS 15: when mild TBI isn’t so mild“, an abridged version of the guideline „Cryptogenic stroke and patent foramen ovale“, a SOP for „First-ever epileptic seizure in adult patients“ and a clinical trial protocol for a study comparing pallidal stimulation and botulinum toxin in cervical dystonia.

We will follow up this exciting issue with more articles in the forthcoming months from across the broad range of topics and article types covered in the journal.

I sincerely hope that you find this first set of articles interesting, inspiring and of a high quality. To reach our ambitious goals for the journal, we need your support. Please submit your research to *Neurological Research and Practice* (all APCs are sponsored by the DGN), feel free to give us your ideas for review articles as well as pass on your feedback and, when appropriate, disseminate and cite our articles. As the journal is open access your work will be highly visible and accessible to an international audience.

Best regards.

